# Current Epidemiology and Co-Infections of Avian Immunosuppressive and Neoplastic Diseases in Chicken Flocks in Central China

**DOI:** 10.3390/v14122599

**Published:** 2022-11-22

**Authors:** Lu-Ping Zheng, Man Teng, Gui-Xi Li, Wen-Kai Zhang, Wei-Dong Wang, Jin-Ling Liu, Lin-Yan Li, Yongxiu Yao, Venugopal Nair, Jun Luo

**Affiliations:** 1 Key Laboratory of Animal Immunology, Ministry of Agriculture and Rural Affairs of China & Henan Provincial Key Laboratory of Animal Immunology, Henan Academy of Agricultural Sciences, Zhengzhou 450002, China; 2 UK-China Centre of Excellence for Research on Avian Diseases, Henan Academy of Agricultural Sciences, Zhengzhou 450002, China; 3 Shangqiu Center for Animal Disease Control and Prevention, Shangqiu 476000, China; 4 College of Animal Science and Technology, Henan University of Science and Technology, Luoyang 471003, China; 5 The Pirbright Institute & UK-China Centre of Excellence for Research on Avian Diseases, Pirbright, Ash Road, Guildford, Surrey GU24 0NF, UK

**Keywords:** poultry, avian neoplastic disease, MDV, ALV, REV, epidemiology, co-infection

## Abstract

The avian immunosuppressive and neoplastic diseases caused by Marek’s disease virus (MDV), avian leucosis virus (ALV), and reticuloendotheliosis virus (REV) are seriously harmful to the global poultry industry. In recent years, particularly in 2020–2022, outbreaks of such diseases in chicken flocks frequently occurred in China. Herein, we collected live diseased birds from 30 poultry farms, out of 42 farms with tumour-bearing chicken flocks distributed in central China, to investigate the current epidemiology and co-infections of these viruses. The results showed that in individual diseased birds, the positive infection rates of MDV, ALV, and REV were 69.5% (203/292), 14.4% (42/292), and 4.7% (13/277), respectively, while for the flocks, the positive infection rates were 96.7% (29/30), 36.7% (11/30), and 20% (6/30), respectively. For chicken flocks, monoinfection of MDV, ALV, or REV was 53.3% (16/30), 3.3% (1/30), and 0% (0/30), respectively, but a total of 43.3% (13/30) co-infections was observed, which includes 23.3% (7/30) of MDV+ALV, 10.0% (3/30) of MDV+REV, and 10.0% (3/30) of MDV+ALV+REV co-infections. Interestingly, no ALV+REV co-infection or REV monoinfection was observed in the selected poultry farms. Our data indicate that the prevalence of virulent MDV strains, partially accompanied with ALV and/or REV co-infections, is the main reason for current outbreaks of avian neoplastic diseases in central China, providing an important reference for the future control of disease.

## 1. Introduction

Avian immunosuppressive and neoplastic diseases commonly cause lymphoid tissue hyperplasia, as well as skin or visceral tumours, in chicken hosts, resulting in severe immunosuppression, a significantly decreased production performance, and large numbers of chicken tumours and deaths, which have led to huge economic losses to the global poultry industry every year. Many pathogens, such as Marek’s disease virus (MDV), avian leukosis virus (ALV), reticuloendotheliosis virus (REV), and their co-infections in chicken flocks are the main factors causing avian neoplastic diseases in poultry [1,2]. Among these diseases, Marek’s disease (MD) caused by MDV is the only one that can be successfully prevented and controlled by vaccination using avirulent or attenuated MD vaccines. However, in recent years, because of the persistent increased virulence and genovariations of epidemic strains and MDV variants, immunization failure of MD has often occurred, which has brought a new challenge to the current prevention and control of disease [3,4,5]. MDV mainly infects chickens and leads to immunosuppression, characterized by serious atrophy of immune organs at an early stage, and induces omasums, slow growth, neurological symptoms, and skin or visceral tumours in clinical diseased birds at a late stage. Usually, it causes a mortality of 10%–60% and an economic loss of more than 1 billion of US dollars annually [6]. Thus, for the control of MD, it is important to monitor the epidemiology of MDV, isolate epidemic strains, and reveal its biological features. According to the latest virus classification and taxon nomenclature [7], the commonly known MDV belongs to the genus *Mardivirus* of the subfamily *Alphaherpesvirinae* and has been reclassified into three types: MDV type 1 (MDV-1) or *Gallid alphaherpesvirus* 2 (GaAHV-2), MDV type 2 (MDV-2) or *Gallid alphaherpesvirus* 3 (GaAHV-3), and herpesvirus of turkey (HVT) or *Meleagrid alphaherpesvirus* 1 (MeAHV-1). However, only the virulent strains of MDV-1 are pathogenic and oncogenic to chicken hosts. As a major oncogene, the Meq (MDV EcoRI-Q) gene is specific for MDV-1 and is closely related to the virulence and pathogenicity of MDV [8]. Therefore, except for being used for phylogenetic analysis to investigate the genetic evolution of MDV [9,10,11], Meq is also regarded as an important diagnostic marker for differentiating MDV-1 epidemic strains from those of avirulent or attenuated MD vaccines.

Avian leukosis (AL) caused by ALV is a vertically-transmitted disease that results in severe immunosuppression, multiple organ retardation, atrophy, and tumours in chickens, and leads to a serious decline in performance and a large number of deaths [1]. ALV belongs to the genus *Alpharetrovirus* of the subfamily *Orthoretrovirinae* [12] and can be divided into seven subtypes, including ALV-A, ALV-B, ALV-C, ALV-D, ALV-E, ALV-J, and ALV-K. Clinically, three subtypes of ALV-A, ALV-B, and ALV-J are more commonly occurring in broilers, layers, breeders, and turkeys [13,14,15,16]. However, to date, no effective vaccine or specific drugs are available for the control of AL, except for the eradication of ALV to block its vertical transmission and spread in chicken flocks. Considering the high economic loss and time spent on ALV eradication, a strengthened real-time epidemiological surveillance of ALV is important for preventing the prevalence of ALV and consolidating the effect of ALV eradication. A variety of methods and diagnostic reagents, such as enzyme-linked immunosorbent assay (ELISA) kits and colloidal gold conjugated immunochromatographic strips, are commonly used for the rapid detection of ALV in anal swabs, meconium, or egg white samples of suspected diseased birds [17,18]. Importantly, it has been demonstrated that isolation of the peripheral blood leukocytes (PBLs) from diseased birds for cultivation on host cells, and furthermore, the detection of the culture supernatant, are the gold standard for ALV detection [19].

Reticuloendotheliosis (RE), caused by REV, which belongs to the genus *Gammaretrovirus* of the subfamily *Orthoretrovirinae* [12], is another harmful vertically transmitted infectious pathogen inducing immunosuppression, acute fatal reticuloendothelioma, short stature syndrome, and chronic tumours in poultry [1]. Usually, monoinfection of REV does not present apparent clinical symptoms and is mostly ignored. Once co-infected with other pathogens, especially with MDV and ALV-J, it shows obvious clinical symptoms and immunosuppressions, increases the incidence of tumours in infected chickens, and reduces the efficacy of other vaccines, which finally leads to more serious economic losses [20,21]. Currently, no effective vaccine is available for the prevention and control of REV either. Thus, timely monitoring of single or co-infections of REV is also of great practical significance for the control of avian immunosuppressive and neoplastic diseases.

As one of the biggest poultry breeding bases in the world, the prevalence of infectious diseases in chicken flocks has seriously affected the healthy development of poultry industry in China. In recent years, the prevalence and co-infections of tumour-related pathogens in large-scale poultry farms in China have become increasingly serious [22,23,24]. Particularly in 2020–2022, outbreaks of avian neoplastic diseases have frequently occurred in many poultry farms in China, from the South to North. Herein, we have collected live diseased birds from 30 poultry farms, out of 42 farms with tumour-bearing chicken flocks distributed in central China, and performed a systematic epidemiological investigation on the prevalence and co-infections of MDV, ALV, and REV in chicken flocks. Our data have demonstrated that the prevalence of virulent MDV strains, partially accompanied with co-infections of ALV and/or REV, is the main reason for current outbreaks of avian neoplastic diseases in central China, providing an important reference for farmers to take timely and effective measures to control such diseases.

## 2. Materials and Methods

### 2.1. Ethics Statement 

Sample collection from a total of 292 clinical diseased birds was performed according to the protocols of the Laboratory Animal-Guidelines for Ethical Review of Animal Welfare, permitted by the State Administration for Market Regulation and Standardization Administration of China (permit no. GB/T 35892-2018). The experimental protocols were approved by the Laboratory Animal Management Committee of Key Laboratory of Animal Immunology, Ministry of Agriculture and Rural Affairs, the People’s Republic of China.

### 2.2. Viruses and Cells

The vvMDV strain Md5 [25] (gift from Prof. Zhi-Zhong Cui, Shandong Agricultural University, China) and a Chinese MDV/REV co-infection strain HNGS206 [9,26] served as positive controls. The specific pathogen free (SPF) eggs were provided by Beijing Boehringer Ingelheim Vital Biotechnology Co., Ltd. (China), and the primary chicken embryo fibroblast (CEF) cells were prepared from 9-day-old embryos. The CEFs were maintained in Dulbecco’s Modified Eagle’s Medium (DMEM) (Gibco, USA) containing 5% fetal bovine serum (FBS) (Sigma, USA), and penicillin–streptomycin (Ncmbio, China), and incubated at 37 °C in a 5% CO_2_ incubator.

### 2.3. Sample Collection

**Figure 1:**
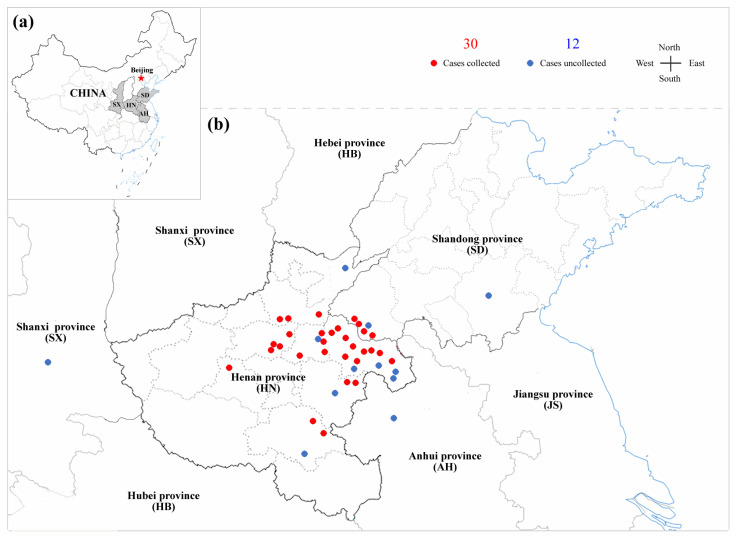
Distribution of poultry farms with neoplastic disease cases in chicken flocks selected from central China during 2020–2022. (**a**) Geographic location of central provinces in China. (**b**) Poultry farms with case reports in central China. All of the cases selected, sampled (2020–2021) or unsampled (2022), are shown by red and blue spots, respectively.

During 2020–2022, outbreaks of suspected avian neoplastic diseases in chicken flocks of 42 poultry farms distributed in provinces of central China, as demonstrated in Figure 1, were initially diagnosed as neoplastic diseases with tumours by local veterinarians. The live diseased birds from 30 poultry farms were used for sampling. The background of poultry farms and detailed information of selected chicken flocks are shown in Table 1. For each bird, 1–2 mL of anticoagulant blood was collected from the inferior pterygeal vein for the isolation of PBLs, and an anal swab was simultaneously collected and placed into a pre-cooled EP tube with 1 mL PBS on ice. Then, the birds were humanly euthanized and necropsy was performed to collect the liver and spleen tissues. All of the tissues and anal swab samples were immediately frozen at −20 °C for further experiments.

### 2.4. Virus Isolation 

According to the instructions of the Lymphocyte Separation Kit (Chicken) (P8740, Solarbio, China), PBLs were isolated from the anticoagulant blood samples collected from each bird and separately inoculated into CEF monolayers on 6-well plates and cultivated for 3 h at 37 °C in a 5% CO_2_ incubator. The supernatant was discarded, refreshed with 2% FBS DMEM medium and maintained for a further 3–5 days, followed by digestion with 0.25% trypsin, frozen and thawed, and two more times of blind passages on fresh CEF monolayers for 5–7 days. For each passage, the cell cultures were collected, frozen and thawed three times, and finally stored at −20 °C for further examinations.

### 2.5. PCR 

The cellular DNA was extracted from the liver and spleen tissue samples and PBL cell cultures using a TIANamp Genomic DNA Kit (DP304, Tiangen Biotech, China). The amplification of the MDV-1 specific Meq gene was performed by polymerase chain reactions (PCR), as previously described using the primers listed in Table 2 [9]. The DNA extracted from Md5-infected CEF cells served as a positive control. The PCR products were analysed by 1% agarose gel electrophoresis and the target band of 1020 bp will be expected from the positive MDV infection. If the positive band was detected from any of the liver, spleen, and PBL cell culture samples from a same bird, the chicken was designated as an MDV infected bird and the corresponding chicken flock/poultry farm was identified as confirmed MD positive flock/farm. 

### 2.6. RT-PCR 

The conventional reverse transcription PCR (RT-PCR) was performed to detect the infection of REV. Briefly, the total cellular RNA from the liver, spleen, and PBL cell cultures were separately extracted using the MiniBEST Universal RNA Extraction Kit (9767, TaKaRa, China). The cDNA was synthesized using a TransScript^®^ One-Step gDNA Removal and cDNA Synthesis SuperMix (AT311, TransGen Biotech, China) and using the primers listed in Table 2. The amplifications of REV LTR, gag, and pol genes by PCR were performed as previously described [26]. The viral RNA extracted from HNGS206-infected CEF cells served as a positive control. If any of the three REV target genes, with a length of 367 bp, 309 bp, or 475 bp, were amplified as expected, it was considered as a positive infection of REV. Once any of the liver, spleen, or PBL cell cultures from the same bird tested positive, the chicken was considered to be an REV-positive infected bird, of which the original chicken flock/poultry farm was identified to be positive for REV infection.

### 2.7. ELISA and Strip Tests

The ELISA kit and colloidal gold immunochromatographic strips were used for the detection of ALV infection in clinical cases. Briefly, the anal swabs collected from clinical diseased birds and PBL cell cultures were freeze–thawed three times and centrifuged at 8000 rpm for 10 min, and then 100 μL of each supernatant was sampled for the detection of ALV using the Avian Leukemia Virus Antigen Test Kit (NEE83500, NECVB, China) according to the manufacturer’s instructions. The S/P value was calculated according to formula S/P= (sample OD_630_ value − negative control OD_630_ value)/(positive control OD_630_ value − negative control OD_630_ value). An S/P value of ≥0.2 was considered as being positive, with an S/P value below 0.2 was negative. Furthermore, the treated supernatants of the anal swabs were simultaneously detected using the Colloidal Gold Strips to Detect the Avian Leukemia Virus Group Specific Antigen (NECVB, China), according to the manufacturer’s protocols. A positive result for either the sample of the anal swab or the PBL cell cultures from the same bird were considered to be an ALV-positive infection and the original chicken flock/poultry farm was ALV positive. 

## 3. Results

### 3.1. Clinical Cases of Avian Neoplastic Diseases and Distribution in Central China 

During 2020–2022, a large number of suspected clinical cases of avian neoplastic diseases were reported in China. To reveal the current epidemiology and co-infection of the prevalent pathogens in chicken flocks, we presently performed a systematic investigation on a total of 30 selected poultry farms, out of 42 farms that have shown suspected diseased birds with gross tumours in chicken flocks. As demonstrated in Figure 1, the selected poultry farms were mainly distributed in central China, and were especially concentrated in the areas near the borders of Henan, Shandong, and Anhui provinces. The diseased flocks from 30 poultry farms, as listed in Table 1, included 26 layer flocks, 2 broiler flocks, and 2 breeder flocks, with a variety of breeds such as Hyline Brown, Jinghong, Liangfenghua, Partridge chicken, and Muyuan Red. For direct examination or virus isolation, a total of 1042 clinical samples from diseased birds were collected, including 292 liver tissue samples, 292 spleen tissue samples, 176 anticoagulant blood samples, and 282 anal swab samples.

### 3.2. Infection Status of MDV, ALV, and REV in Birds with Suspected Neoplastic Diseases 

**Figure 2:**
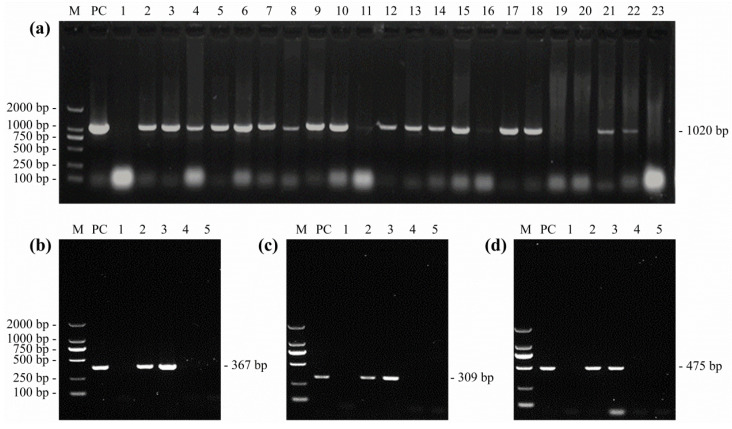
Amplification of MDV and REV specific genes from clinical samples. Amplification of the MDV-1 specific Meq gene by PCR (**a**) and the amplification of REV LTR, gag, or pol genes by RT-PCR (**b**–**d**). M, DNA marker; PC, positive control; lines 1–23 or 1–5, numbers of detected clinical samples. Because of space limitations, only part of data are shown here.

The PCR and RT-PCR analyses were separately performed to detect the MDV or REV in the livers, spleens, and PBL cell cultures derived from clinically diseased birds. The results have shown that the specific amplicons of MDV-1 Meq genes in 1020 bp in length were observed in the PCR products of most of the detected samples, while the REV LTR, gag, and pol genes, with sizes of 367 bp, 309 bp, and 475 bp, respectively, were only amplified from a small number of samples (Figure 2). Simultaneously, the anal swab samples and PBL cell cultures were subject to both ELISA kit and test strips for the detection of ALV P27 antigens. A summary of the detailed detection results of the monoinfection of MDV, ALV, and REV in 1042 samples collected from 30 chicken flocks in different poultry farms is shown in Table S1. The result demonstrates that for individual diseased birds, the positive detection rates of MDV, ALV, and REV infection were 69.5% (203/292), 14.4% (42/292), and 4.7% (13/277), respectively. For chicken flocks, a total of 29, 11, and 6 poultry farms were found to be positively infected with MDV, ALV, and REV, with a positive rate of 96.7%, 36.7%, and 20%, respectively. 

### 3.3. Co-Infections of MDV, ALV, and REV in Chicken Flocks

**Figure 3:**
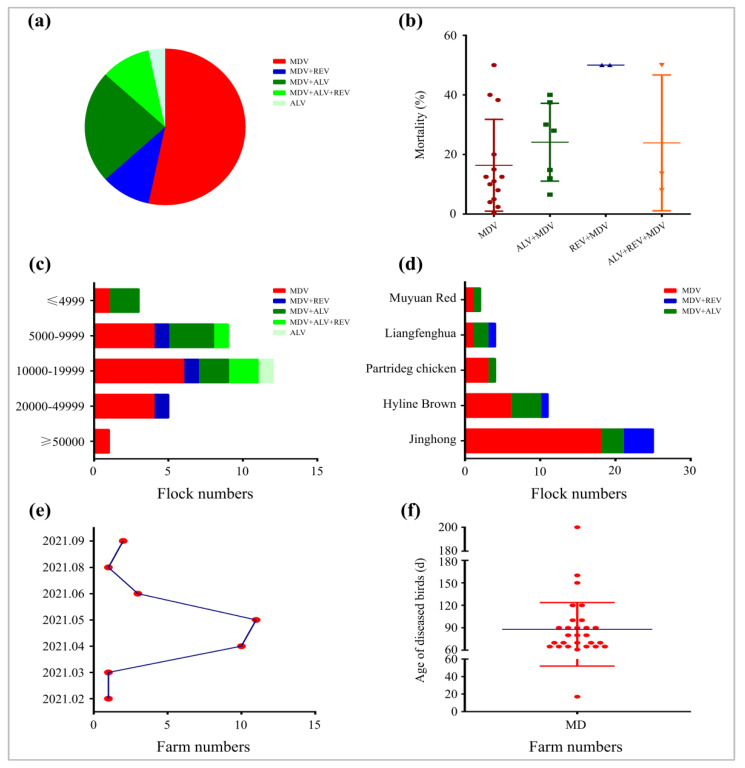
Epidemiological data analysis of MDV, ALV, and REV infections in chicken flocks selected from 30 poultry farms. (**a**) Distribution schematic of mono or co-infections of MDV, ALV, and/or REV in chicken flocks. (**b**) Mortality of chickens infected with MDV or co-infected with different combination of viruses. (**c**) Distribution of three pathogens in different sizes of chicken flocks. (**d**) Distribution of three pathogens in different chicken breeds. (**e**) Numbers of case reports at different time points. (**f**) Statistic of the onset age of disease for MD cases.

Among all of the 30 tested chicken flocks, as shown in Table 3 and Figure 3a, 53.3% (16/30), 3.3% (1/30), and 0% (0/30) of them were monoinfected with MDV, ALV, or REV, respectively. Co-infection of MDV with ALV and/or REV was commonly observed in poultry farms (Table 3), of which the co-infection rates of MDV+ALV, MDV+REV, and MDV+ALV+REV were 23.3% (7/30), 10.0% (3/30), or 10.0% (3/30), respectively. However, no ALV+REV co-infection or REV infection alone were found in any of the 30 cases. However, compared with the monoinfection of MDV, co-infection with any of the other two pathogens (ALV and/or REV) has without exception increased the mortality of diseased birds (Table 1 and Figure 3b).

### 3.4. Relationship between Breeding Scale, Chicken Breeds, and Virus Infection 

For all of the investigated 30 poultry farms, the chicken flocks were divided into five groups based on the size of the flocks: less than 5000 birds, 5000–9999 birds, 10,000–19,999 birds, 20,000–49,000 birds, and more than 50,000 birds. As demonstrated in Figure 3c, MDV infection could be detected in chicken flocks regardless of the size of the poultry farm. However, in smaller sized chicken flocks with less than 20,000 birds, co-infections of two viruses of MDV+ALV or MDV+REV, and even three viruses of MDV+ALV+REV, were commonly detected. It seems that co-infection of MDV with the other two viruses apparently decreased with the increased size of chicken flocks (Figure 3c). Interestingly, among all of the five tested chicken breeds, including Hyline Brown, Jinghong, Liangfenghua, Partridge chicken, and Muyuan Red, infections of MDV, ALV, and REV were commonly observed, regardless of the breeds (Table 3 and Figure 3d). For Chinese local breeds, such as Liangfenghua, Partridge chicken and Muyuan Red, some of the poultry farms had a high proportion of ALV infection, while for the larger commercial breeds such as Hyline Brown and Jinghong, ALV infection was negative in the majority of chicken flocks. 

### 3.5. Seasonal and Age Features Correlated to Current MD Outbreaks 

Among the 30 poultry farms tested, a total of 29 chicken flocks were finally diagnosed as being MD positive, although nearly half of them were co-infected with ALV and/or REV. For the time point of the case report, it has been observed that the occurrence of MD cases was quickly increased from March to April and peaked in May in 2021 (Figure 3e). During this time period, MD cases were reported in 23 chicken flocks, including 10 cases in April and 11 cases in May (Table 3). This may be closely related to the seasonal breeding habits in poultry production in China, which hatches chicks in early spring, and consequentially the occurrence of MD cases mainly happens in late spring and early summer every year. In addition, based on the collected data of the onset age of disease, as shown in Table 1 and Figure 3f, the onset days of MD cases with tumours mainly ranged from 60–120 days, with a median age of 90 days. However, out of our expectation, the occurrence of MD cases at the lowest age of 17 days in broiler chicks or at the highest age of 200 days in layer hens were both observed.

## 4. Discussion

In recent years, outbreaks of avian immunosuppressive and neoplastic diseases such as MD, AL, and RE have been frequently reported in chicken flocks worldwide, including in China [2,9,10,11,13,14,15,20,21,22,23,27,28,29,30]. Especially during 2020–2022, a concentrated outbreak of suspected neoplastic diseases occurred in poultry farms from the South to North of China, but the main reasons remained unclear. Thus, in the present study, we performed an overall investigation on the epidemiology of the potential pathogens prevalent in poultry farms distributed in central China. Our data revealed that in chicken flocks with diseased birds, the infection rates of MDV were significantly higher than that of ALV and REV, which came from the analysis of the corresponding individual clinical infection data of MDV, ALV, and REV, respectively. The monoinfection of MDV, ALV, or REV in chicken flocks was observed to be only a bit higher than half of the cases, while the double or triple co-infections accounted for nearly another half, which included co-infections of MDV+ALV, MDV+REV, and even MDV+ALV+REV. However, no REV monoinfection and ALV+REV co-infection were observed in all of the selected 30 chicken flocks from poultry farms with clinical cases. For most cases, high mortalities were observed in chicken flocks co-infected with two or three pathogens. However, for some cases, only a relatively lower mortality occurred. This may be because the mortality listed in the background was not the final statistics of these chicken flocks, but only provided by the farmers on the time points of our sample collections. The positive flocks were judged by any kind of virus infection in an individual bird, but it does not mean the co-infections occurred in a same bird, which certainly could not completely reflect the total mortality of a poultry farm. The co-infection and secondary infection of other avian pathogens may further enhance the mortality and seriousness of tumour-bearing chicken flocks. For further studies, to give an overview on the potential pathogenic factors in chicken flocks with diseased birds, we need to detect more avian pathogens, such as immunosuppression agents, including infection bursal disease virus (IBDV), chicken infectious anemia virus (CIAV), and avian reovirus (ARV), as well as hepatosplenomegaly pathogens, including avian hepatitis E virus (HEV) and fowl adenovirus (FAdV) that can cause similar symptoms or lesions to MDV, ALV, and REV. In conclusion, the data from in this study indicate that the prevalence of virulent MDV, partially accompanied with ALV and/or REV co-infections, is the main reason for current outbreaks of avian neoplastic diseases in central China. 

Previously, an investigation on the prevalence of ALV-J and REV in 29 chicken flocks of various commercial and local breeds in six provinces in China from 1999 to 2009 was conducted and the results showed that REV was positively isolated from 19 flocks, and one-third of ALV-J isolates (11/32 from 8/15 flocks) were co-infected with REV [31]. Another survey on 480 chicken plasma samples collected from both of the parental and protospecies flocks demonstrated that the positive rate of REV infection was 22.29%, which is the main immunosuppressive virus co-infected with fowl adenovirus (FAdV) to cause serious decreased egg production, including body hepatitis and pericardial effusion syndrome in chickens [32]. A recent epidemiological study on a total of 1230 samples (1144 feather pulps and 86 PBLs) from 305 chicken flocks collected from 12 provinces in China from 2011 to 2015 demonstrated that among all of the MDV-positive samples, co-infection of REV was positively detected in 13.0% (79/606) of samples from 18.8% (31/165) chicken flocks, and the subsequent animal experiments showed that the co-infection of REV significantly promoted both of the mortality and tumour occurrence of MDV isolates [33]. All of these studies, together with our present data, suggest that currently in China, the infection of REV alone is probably not a key factor, but its secondary infection with MDV or ALV usually results in increased seriousness of avian oncogenic and tumour diseases. 

Co-infection of ALV with MDV also can lead to increased viral replication, enhanced pathogenicity, and the occurrence of tumours in chicken [34,35]. Presently, we also observed that the average mortality of most chickens co-infected with MDV, ALV, and/or REV was higher than those chickens infected with MDV alone. During 2015–2017, a total of 2509 laboratory diagnosis reports from eight states in USA were studied and the investigators found that tumour or lymphoproliferative diseases accounted for 42% of all poultry cases examined at autopsy, and 63% of them were diagnosed as MD or AL [36]. This indicates that avian neoplastic diseases such as MD and AL are not only harmful to the poultry industry in Asia, but also in North America. In the past decade, with the large-scale eradication of ALV in China, incidence of ALV in some commercial chicken breeds such as Hyline Brown and white-feathered broilers has been greatly reduced. However, some local breeds in China still have a high incidence [37,38]. In this study, ALV infection was found in all of the five different breeds of chickens, including three Chinese local chicken species with a higher positive infection rate, suggesting that the eradication of ALV particularly in local chickens should be strengthened in the future. The two methods, namely the ELISA kit and test strip, presently used for the detection of the P27 antigen, cannot distinguish the exogenous and endogenous ALV. However, for most positive flocks, typical clinical symptoms of AL, including abdominal enlargement, big liver tumours, protuberances on shanks, and hemangiomas appearing as blood blisters, were observed in diseased birds from our background investigations (data not shown). Thus, this provided useful data for evaluating the status of virus infection and the potential risk in poultry farms. For future work, the subtypes of exogenous epidemic ALV strains should be further investigated through virus isolation on DF-1 cells, which can exclude the growth of the endogenous virus. Obviously, it is extremely important to carry out a long-term and continuous ALV eradication in both conventional and local breeds of chickens based on a real time epidemiological monitoring for the effective prevention and control of such a poultry tumour disease.

As discussed above, the prevalence and infection of MDV is the key pathogenic factor responsible for current outbreaks of avian neoplastic diseases in central China. However, except for three poultry farms, all of the other investigated farmers and providers stated that all the diseased birds had been vaccinated at one-day old with MD vaccines, although the detailed vaccine strains and producers of the commercial MD vaccines were unavailable. Many factors may cause the immune failure of MD vaccination, including the potential vaccine quality problems, storage and transportation of the vaccines, and especially the decreased immune protection caused by the increased virulence and genovariation of MDV circulating strains [3,4,5]. Thus, for the future control of disease, more work such as the isolation of prevalent MDV strains, genetic evolution analysis, evaluation of immune protection of current available MD vaccine products, and development of novel highly efficient vaccines needs to be done. In addition, our data have also demonstrated that for most of the present investigated MD cases, the concentrated age of disease ranged from 60–120 days, with a median age of 90 days. However, several clinical MD cases outbreak in 17-day-old broiler chicks and 200-day old layer hens at the peak of the egg-laying period have been presently observed. In a previous study [39], a clinical case of MD in a 24–30-week-old vaccinated broiler breeder flock was reported in China. Combined with the present findings, it is obvious that the period of onset of MD in chickens has expanded, which has brought a new challenge for future study and control of the disease.

## Figures and Tables

**Table 1:** Background of clinical cases of avian neoplastic diseases from poultry farms distributed in central China.

No.	Poultry Farms	Breeds	Category	Geographical Location *	Bird Nos.	Age for Sample Collection (Days)	Mortality	Year & Month
1	HNZMD	Liangfenghua	Broiler	Henan, Zhumadian	5000	120	50.0%	2020, November
2	HNXZ1	Partridge chicken	Layer	Henan, Xinzheng	60,000	90	50.0%	2021, February
3	HNZM	Partridge chicken	Broiler	Henan, Zhongmu	8000	17	37.5%	2021, March
4	HNXZ2	Liangfenghua	Breeder	Henan, Xinzheng	15,000	190	UA	2021, April
5	HNYY1	Jinghong	Layer	Henan, Yuanyang	15,000	90	50.0%	2021, April
6	HNLK1	Hyline Brown	Layer	Henan, Lankao	20,000	150	12.5%	2021, April
7	HNLK2	Jinghong	Layer	Henan, Lankao	15,000	160	5.0%	2021, April
8	HNZC1	Jinghong	Layer	Henan, Zhecheng	20,000	90	38.3%	2021, April
9	HNSQ1	Jinghong	Layer	Henan, Shangqiu	16,800	100	40.0%	2021, April
10	HNLY1	Jinghong	Layer	Henan, Luyi	42,000	120	50.0%	2021, April
11	HNYC1	Jinghong	Layer	Henan, Yucheng	7000	61	10.0%	2021, April
12	SDCX1	Jinghong	Layer	Shandong, Caoxian	2000	100	30.0%	2021, April
13	SDSX	Jinghong	Layer	Shandong, Shanxian	10,000	65	15.0%	2021, April
14	SDCW	Hyline Brown	Layer	Shandong, Chengwu	10,000	70	20.0%	2021, May
15	SDCX2	Jinghong	Layer	Shandong, Caoxian	5400	80	6.50%	2021, May
16	HNSQ2	Jinghong	Layer	Henan, Shangqiu	9000	70	11.0%	2021, May
17	HNSC	Hyline Brown	Layer	Henan, Shangcai	11,000	80	13.6%	2021, May
18	HNYC2	Jinghong	Layer	Henan, Yucheng	5200	65	28.0%	2021, May
19	HNZC2	Jinghong	Layer	Henan, Zhecheng	11,000	70	8.0%	2021, May
20	HNXZ3	Partridge chicken	Breeder	Henan, Xinzheng	20,000	80	4.0%	2021, May
21	HNQX	Jinghong	Layer	Henan, Qixian	10,000	65	12.5%	2021, May
22	HNZC3	Jinghong	Layer	Henan, Zhecheng	2800	65	UA	2021, May
23	HNYY2	Jinghong	Layer	Henan, Yuanyang	44,000	65	2.4%	2021, May
24	HNPDS	Hyline Brown	Layer	Henan, Pingdingshan	5000	65	12.0%	2021, May
25	HNLY2	Jinghong	Layer	Henan, Luyi	17,000	90	0.3%	2021, June
26	HNZC4	Hyline Brown	Layer	Henan, Zhecheng	2700	90	14.8%	2021, June
27	HNSX	Jinghong	Layer	Henan, Shanxian	8000	90	8.0%	2021, June
28	HNZC5	Jinghong	Layer	Henan, Zhecheng	6000	200	UA	2021, August
29	HNFQ	Hyline Brown	Layer	Henan, Fengqou	12,000	70	UA	2021, September
30	HNWS	Muyuan Red	Layer	Henan, Weishi	18,000	70	40.0%	2021, September

* Geographical location is displayed as province plus city/county. UA, unavailable.

**Table 2:** Primers for amplifying the viral genes of MDV and REV used in this study.

Virus	Target	Primer	Sequences ( 5′-3′)	Amplicon (bp)
MDV	meq	MDV-meq-F	ATGTCTCAGGAGCCAGAG	1020
		MDV-meq-R	TCAGGGTCTCCCGTCACC	
REV	LTR	REV-LTR-F	CATGCTTGCTTGCCTTAGC	367
		REV-LTR-R	CCTCTCACTGCCAATCTGAG	
	gag	REV-gag-F	TCAGGCTGCCATAGTCATTC	309
		REV-gag-R	TTCTTCTTCCAATGTCCCTC	
	pol	REV-pol-F	AGCCTCTAAACTTACCTTCG	475
		REV-pol-R	GTTGACGCTCTTGTCCTTGC	

**Table 3:** Co-infections of three pathogens in chickens with suspected neoplastic disease from poultry farms distributed in central China.

No.	Poultry Farms	Breeds	Category	Positive rates of Three Pathogens			Diagnosis Results ^#^						
				MDV	ALV	REV	MDV	ALV	REV	M+A	M+R	A+R	M+A+R
1	HNZMD	Liangfenghua	Broiler	100% (12/12)	100% (6/6)	100% (6/6)							*
2	HNXZ1	Partridge chicken	Layer	100% (6/6)	0% (0/6)	0% (0/6)	*						
3	HNZM	Partridge chicken	Broiler	33.3% (4/12)	16.7% (2/12)	0% (0/12)				*			
4	HNXZ2	Liangfenghua	Breeder	0% (0/7)	71.4% (5/7)	0% (0/7)		*					
5	HNYY1	Jinghong	Layer	86.7% (13/15)	0% (0/15)	6.7% (1/15)					*		
6	HNLK1	Hyline Brown	Layer	20% (3/15)	0% (0/15)	0% (0/15)	*						
7	HNLK2	Jinghong	Layer	12.5% (1/8)	0% (0/15)	0% (0/8)	*						
8	HNZC1	Jinghong	Layer	50% (3/6)	0% (0/6)	0% (0/6)	*						
9	HNSQ1	Jinghong	Layer	92.7% (11/12)	0% (0/12)	0% (0/12)	*						
10	HNLY1	Jinghong	Layer	75% (3/4)	0% (0/4)	25% (1/4)					*		
11	HNYC1	Jinghong	Layer	100% (14/14)	0% (0/14)	0% (0/12)	*						
12	SDCX1	Jinghong	Layer	100% (9/9)	22.2% (2/9)	0% (0/9)				*			
13	SDSX	Jinghong	Layer	54.5% (6/11)	0% (0/11)	0% (0/11)	*						
14	SDCW	Hyline Brown	Layer	94.1% (16/17)	0% (0/17)	0% (0/17)	*						
15	SDCX2	Jinghong	Layer	100% (8/8)	25% (1/4)	0% (0/4)				*			
16	HNSQ2	Jinghong	Layer	80% (4/5)	0% (0/2)	0% (0/2)	*						
17	HNSC	Hyline Brown	Layer	100% (14/14)	14.3% (2/14)	21.4% (3/14)							*
18	HNYC2	Jinghong	Layer	100% (14/14)	0% (0/14)	7.1% (1/14)					*		
19	HNZC2	Jinghong	Layer	92.3% (12/13)	15.4% (2/13)	7.7% (1/13)							*
20	HNXZ3	Partridge chicken	Breeder	87.5% (7/8)	0% (0/9)	0% (0/8)	*						
21	HNQX	Jinghong	Layer	100% (9/9)	0% (0/10)	0% (0/9)	*						
22	HNZC3	Jinghong	Layer	40% (2/5)	0% (0/5)	0% (0/5)	*						
23	HNYY2	Jinghong	Layer	40% (2/5)	0% (0/5)	0% (0/5)	*						
24	HNPDS	Hyline Brown	Layer	16.7% (1/6)	16.7% (1/6)	0% (0/6)				*			
25	HNLY2	Jinghong	Layer	100% (6/6)	0% (0/6)	0% (0/6)	*						
26	HNZC4	Hyline Brown	Layer	100% (8/8)	12.5% (1/8)	0% (0/8)				*			
27	HNSX	Jinghong	Layer	100% (5/5)	0% (0/5)	0% (0/5)	*						
28	HNZC5	Jinghong	Layer	71.4% (5/7)	0% (0/7)	0% (0/7)	*						
29	HNFQ	Hyline Brown	Layer	18.2% (2/11)	18.2% (2/11)	0% (0/11)				*			
30	HNWS	Muyuan Red	Layer	15% (3/20)	90% (18/20)	0% (0/20)				*			
Total	NA	NA	NA	69.5% (203/292)	14.4% (42/292)	4.7% (13/277)	53.3% (16/30)	3.3% (1/30)	0% (0/30)	23.3% (7/30)	10.0% (3/30)	0% (0/30)	10.0% (3/30)

* Positive infection or co-infection of MDV, ALV and/or REV. ^#^ M+A, MDV+ALV; M+R, MDV+REV; A+R, ALV+REV; M+A+R, MDV+ALV+REV. NA, not applicable.

## Data Availability

Not applicable.

## Supplementary Materials

The following supporting information can be downloaded at: https://www.mdpi.com/article/10.3390/v14122599/s1. Table S1: Detail data of three pathogen infections in chickens with suspected neoplastic diseases collected from poultry farms distributed in central China.

## References

[1] Swayne D.E., Boulianne M., Logue C.M., McDougald L.R., Nair V., Suarez D.L. (2020). Diseases of Poultry.

[2] Davidson I., Borenshtain R. (2001). In vivo events of retroviral long terminal repeat integration into Marek’s disease virus in commercial poultry detection of chimeric molecules as a marker. Avian Dis..

[3] Teng M., Zheng L.P., Li H.Z., Ma S.M., Zhu Z.J., Chai S.J., Yao Y., Nair V., Zhang G.P., Luo J. (2022). Pathogenicity and Pathotype Analysis of Henan Isolates of Marek’s Disease Virus Reveal Long-Term Circulation of Highly Virulent MDV Variant in China. Viruses..

[4] Song B.L., Zeb J., Hussain S., Aziz M.U., Circella E., Casalino G., Camarda A., Yang G., Buchon N., Sparagano O. (2022). A Review on the Marek’s Disease Outbreak and Its Virulence-Related meq Genovariation in Asia between 2011 and 2021. Animals..

[5] Witter R.L. (1997). Increased Virulence of Marek’s Disease Virus Field Isolates. Avian Dis..

[6] Kennedy D.A., Cairns C., Jones M.J., Bell A.S., Salathe R.M., Baigent S.J., Nair V.K., Dunn P.A., Read A.F. (2017). Industry-Wide Surveillance of Marek’s Disease Virus on Commercial Poultry Farms. Avian Dis..

[7] Gatherer D., Depledge D.P., Hartley C.A., Szpara M.L., Vaz P.K., Benko M., Brandt C.R., Bryant N.A., Dastjerdi A., Doszpoly A. (2021). ICTV Virus Taxonomy Profile: Herpesviridae 2021. J. Gen. Virol..

[8] Conradie A.M., Bertzbach L.D., Trimpert J., Patria J.N., Murata S., Parcells M.S., Kaufer B.B. (2020). Distinct polymorphisms in a single herpesvirus gene are capable of enhancing virulence and mediating vaccinal resistance. PLoS Pathog..

[9] Yu Z.H., Teng M., Luo J., Wang X.W., Ding K., Yu L.L., Su J.W., Chi J.Q., Zhao P., Hu B. (2013). Molecular characteristics and evolutionary analysis of field Marek’s disease virus prevalent in vaccinated chicken flocks in recent years in China. Virus Genes..

[10] Zhang Y.P., Lv H.C., Bao K.Y., Gao Y.L., Gao H.L., le Qi X., Cui H.Y., Wang Y.Q., Li K., Gao L. (2016). Molecular and pathogenicity characterization of Gallid herpesvirus 2 newly isolated in China from 2009 to 2013. Virus Genes..

[11] Deng Q.M., Shi M.Y., Li Q.H., Wang P.K., Li M., Wang W.W., Gao Y.L., Li H.J., Lin L.L., Huang T. (2021). Analysis of the evolution and transmission dynamics of the field MDV in China during the years 1995-2020, indicating the emergence of a unique cluster with the molecular characteristics of vv+ MDV that has become endemic in southern China. Transbound. Emerg. Dis..

[12] Coffin J., Blomberg J., Fan H., Gifford R., Hatziioannou T., Lindemann D., Mayer J., Stoye J., Tristem M., Johnson W. (2021). ICTV Virus Taxonomy Profile: Retroviridae 2021. J. Gen. Virol..

[13] Zhang G.H., Qu Y.J., Niu Y.J., Zhang H.X., Sun Q.Q., Liu X.P., Li Y., Zhang H., Liu M.D. (2019). Difference in pathogenicity of 2 strains of avian leukosis virus subgroup J in broiler chicken. Poult. Sci..

[14] Meng F.F., Li Q.C., Zhang Y.B., Cui Z.Z., Chang S., Zhao P. (2018). Isolation and characterization of subgroup J Avian Leukosis virus associated with hemangioma in commercial Hy-Line chickens. Poult. Sci..

[15] Shao H.X., Wang L., Sang J.J., Li T.F., Liu Y.L., Wan Z.M., Qian K., Qin A.J., Ye J. (2017). Novel avian leukosis viruses from domestic chicken breeds in mainland China. Arch. Virol..

[16] Zeghdoudi M., Aoun L., Merdaci L., Bouzidi N. (2017). Epidemiological features and pathological study of avian leukosis in turkeys’ flocks. Vet. World..

[17] Yu M.M., Bao Y.L., Wang M.P., Zhu H.B., Wang X.Y., Xing L.X., Chang F.F., Liu Y.Z., Farooque M., Wang Y.Q. (2019). Development and application of a colloidal gold test strip for detection of avian leukosis virus. Appl. Microbiol. Biotechnol..

[18] Zhou X.Y., Wang L., Shen A.N., Shen X., Xu M.R., Qian K., Shao H.X., Yao Y.X., Nair V., Ye J.Q. (2019). Detection of ALV p27 in cloacal swabs and virus isolation medium by sELISA. BMC Vet. Res..

[19] Wang X.Z., Wang B., Zhang P.P., Cheng H.G., Song S.H. (2013). The passage of cells can improve the detection rate of avian leukosis virus to facilitate the elimination of avian leukosis in chickens. SpringerPlus..

[20] Sun G.R., Zhang Y.P., Zhou L.Y., Lv H.C., Zhang F., Li K., Gao Y.L., Qi X.L., Cui H.Y., Wang Y.Q. (2017). Co-Infection with Marek’s Disease Virus and Reticuloendotheliosis Virus Increases Illness Severity and Reduces Marek’s Disease Vaccine Efficacy. Viruses..

[21] Li L., Zhuang P.P., Cheng Z.Q., Yang J., Bi J.M., Wang G.H. (2020). Avian leukosis virus subgroup J and reticuloendotheliosis virus coinfection induced TRIM62 regulation of the actin cytoskeleton. J. Vet. Sci..

[22] Shi M.Y., Li M., Wang P.K., Wang W.W., Li H.J., Gao Y.L., Lin L., Huang T., Wei P. (2021). An outbreak in three-yellow chickens with clinical tumors of high mortality caused by the coinfection of reticuloendotheliosis virus and Marek’s disease virus: A speculated reticuloendotheliosis virus contamination plays an important role in the case. Poult. Sci..

[23] Li M., Wang P.K., Li Q.H., Deng Q.M., Shi M.Y., Mo M.L., Wei T.C., Huang T., Wei P. (2021). Reemergence of reticuloendotheliosis virus and Marek’s disease virus co-infection in Yellow-Chickens in Southern China. Poult. Sci..

[24] Li M., Xiong H.F., Wu H.W., Hu D.M., Lin Y., Huang X.T., Wang J., Qi K.Z., Liu H.M. (2021). Pathologic Characterization of Coinfection with Histomonas meleagridis, Marek’s Disease Virus, and Subtype J Avian Leukosis Virus in Chickens. Avian Dis..

[25] Witter R.L., Sharma J.M., Fadly A.M. (1980). Pathogenicity of Variant Marek’s Disease Virus Isolants in Vaccinated and Unvaccinated Chickens. Avian Dis..

[26] Su J.W., Teng M., Luo J., Chi J.Q., Yu Z.H., Yu L.L., Zhang G.P. (2016). Co-infection of field Marek’s disease virus with reticuloendotheliosis virus prevalent in vaccinated chicken flocks. Acta Vet. Zootech. Sinica..

[27] Zhang Y.P., Li Z.J., Bao K.Y., Lv H.C., Gao Y.L., Gao H.L., Qi X.L., Cui H.Y., Wang Y.Q., Ren X.G. (2015). Pathogenic characteristics of Marek’s disease virus field strains prevalent in China and the effectiveness of existing vaccines against them. Vet. Microbiol..

[28] Wang P.K., Niu J.R., Xue C., Han Z.Q., Abdelazez A., Zhang X.L. (2020). Two novel recombinant avian leukosis virus isolates from Luxi gamecock chickens. Arch. Virol..

[29] Lin L., Wang P.K., Yang Y.L., Li H.J., Huang T., Wei P. (2017). Full-length genome sequence analysis of four subgroup J avian leukosis virus strains isolated from chickens with clinical hemangioma. Virus Genes..

[30] Xu A.H., Huo C.Y., Zhong Q., Xu M.Y., Yang Y.R., Tian H.Y., Zhang G.Z., Hu Y.X. (2020). Isolation and pathogenicity testing of avian reticuloendotheliosis virus from layer chickens in China. J. Vet. Diagn. Investig..

[31] Cui Z.Z., Sun S.H., Zhang Z., Meng S.S. (2009). Simultaneous endemic infections with subgroup J avian leukosis virus and reticuloendotheliosis virus in commercial and local breeds of chickens. Avian Pathol..

[32] Meng F.F., Dong G.W., Zhang Y.B., Tian S.B., Cui Z.Z., Chang S., Zhao P. (2018). Co-infection of fowl adenovirus with different immunosuppressive viruses in a chicken flock. Poult. Sci..

[33] Zhang Y.P., Yu Z.H., Lan X.G., Zhang F., Wang Q., Li K., Pan Q., Gao Y.L., Qi X.L., Cui H.Y. (2019). A high frequency of Gallid herpesvirus-2 co-infection with Reticuloendotheliosis virusis associated with high tumor rates in Chinese chicken farms. Vet. Microbiol..

[34] Zhou J., Zhao G.L., Wang X.M., Du X.S., Su S., Li C.G., Nair V., Yao Y.X., Cheng Z.Q. (2018). Synergistic Viral Replication of Marek’s Disease Virus and Avian Leukosis Virus Subgroup J is Responsible for the Enhanced Pathogenicity in the Superinfection of Chickens. Viruses..

[35] Wen Y.W., Huang Q., Yang C.C., Pan L., Wang G.J., Qi K.Z., Liu H.M. (2018). Characterizing the histopathology of natural co-infection with Marek’s disease virus and subgroup J avian leucosis virus in egg-laying hens. Avian Pathol..

[36] Cadmus K.J., Mete A., Harris M., Anderson D., Davison S., Sato Y., Helm J., Boger L., Odani J., Ficken M.D. (2019). Causes of mortality in backyard poultry in eight states in the United States. J. Vet. Diagn. Investig..

[37] Meng F.F., Li Q.C., Zhang Y.W., Zhang Z.H., Tian S.B., Cui Z.Z., Chang S., Zhao P. (2018). Characterization of subgroup J avian Leukosis virus isolated from Chinese indigenous chickens. Virol. J..

[38] Xu M., Mu X.H., Qian K., Shao H.X., Yao Y.X., Nair V., Wang J., Ye J.Q., Qin A.J. (2021). Novel mutation of avian leukosis virus subgroup J from Tibetan chickens. Poult. Sci..

[39] Zhuang X.Y., Zou H.T., Shi H.Y., Shao H.X., Ye J.Q., Miao J., Wu G.H., Qin A.J. (2015). Outbreak of Marek’s disease in a vaccinated broiler breeding flock during its peak egg-laying period in China. BMC Vet. Res..

