# Supplementary material for: Current Epidemiology and Co-Infections of Avian Immunosuppressive and Neoplastic Diseases in Chicken Flocks in Central China
Source: Viruses. 2022 Nov 22;14(12):2599. doi: 10.3390/v14122599 (PMC9784009; doi:10.3390/v14122599)
Supplement: Supplementary file 1 [file viruses-14-02599-s001.zip › viruses-1969876-supplementary.pdf]

Supplementary data

Table S1 Detail data of three pathogen infections in chickens with suspected neoplastic diseases collected from poultry farms distributed in central China.

No.	Poultry farms	Breeds	Category	Positive rates of three pathogens										
				MDV				ALV			REV			
				Liver	Spleen	Cell culture	Positive rate	Anal swab	Cell culture	Positive rate	Liver	Spleen	Cell culture	Positive rate
1	HNZMD	Liangfenghua	Broiler	12/12	12/12	6/6	100% (12/12)	0/0	6/6	100% (6/6)	0/0	0/0	6/6	100% (6/6)
2	HNXZ1	Partridge chicken	Layer	6/6	5/6	6/6	100% (6/6)	0/6	0/6	0% (0/6)	0/6	0/6	0/6	0% (0/6)
3	HNZM	Partridge chicken	Broiler	0/12	4/12	4/12	33.3% (4/12)	2/12	2/12	16.7% (2/12)	0/12	0/12	0/12	0% (0/12)
4	HNXZ2	Liangfenghua	Breeder	0/7	0/7	0/7	0% (0/7)	3/7	4/7	71.4% (5/7)	0/7	0/7	0/7	0% (0/7)
5	HNYY1	Jinghong	Layer	4/15	12/15	13/15	86.7% (13/15)	0/15	0/15	0% (0/15)	1/15	0/15	0/15	6.7% (1/15)
6	HNLK1	Hyline	Layer	0/15	2/15	3/5	20% (3/15)	0/15	0/5	0% (0/15)	0/15	0/15	0/5	0% (0/15)
7	HNLK2	Jinghong	Layer	0/8	1/8	1/8	12.5% (1/8)	0/15	0/8	0% (0/15)	0/8	0/8	0/8	0% (0/8)
8	HNZC1	Jinghong	Layer	0/6	2/6	3/6	50% (3/6)	0/6	0/6	0% (0/6)	0/6	0/6	0/6	0% (0/6)
9	HNSQ1	Jinghong	Layer	11/12	11/12	5/5	92.7% (11/12)	0/12	0/5	0% (0/12)	0/12	0/12	0/5	0% (0/12)
10	HNLY1	Jinghong	Layer	2/4	2/4	3/4	75% (3/4)	0/4	0/4	0% (0/4)	0/4	1/4	0/4	25% (1/4)
11	HNYC1	Jinghong	Layer	12/14	7/14	4/5	100%(14/14)	0/14	0/5	0% (0/14)	0/12	0/12	0/5	0% (0/12)
12	SDCX1	Jinghong	Layer	9/9	3/9	4/5	100% (9/9)	2/9	2/5	22.2% (2/9)	0/9	0/9	0/5	0% (0/9)
13	SDSX	Jinghong	Layer	1/11	2/11	2/6	54.5% (6/11)	0/11	0/6	0% (0/11)	0/11	0/11	0/6	0% (0/11)
14	SDCW	Hyline	Layer	13/17	15/17	5/6	94.1% (16/17)	0/17	0/6	0% (0/17)	0/17	0/17	0/6	0% (0/17)
15	SDCX2	Jinghong	Layer	7/8	5/8	4/4	100% (8/8)	0/4	1/4	25% (1/4)	0/4	0/4	0/4	0% (0/4)
16	HNSQ2	Jinghong	Layer	4/5	4/5	1/2	80% (4/5)	0/2	0/2	0% (0/2)	0/2	0/2	0/2	0% (0/2)

17	HNSC	Hyline	Layer	11/14	11/14	4/6	100% (14/14)	2/14	0/6	14.3% (2/14)	2/14	1/14	2/6	21.4% (3/14)
18	HNYC2	Jinghong	Layer	11/14	14/14	5/8	100% (14/14)	0/14	0/8	0% (0/14)	0/14	0/14	1/8	7.1% (1/14)
19	HNZC2	Jinghong	Layer	10/13	11/13	5/5	92.3% (12/13)	2/13	1/5	15.4% (2/13)	1/13	0/13	1/5	7.7% (1/13)
20	HNXZ3	Partridge chicken	Breeder	4/8	4/8	5/5	87.5% (7/8)	0/9	0/5	0% (0/9)	0/8	0/8	0/5	0% (0/8)
21	HNQX	Jinghong	Layer	6/9	9/9	5/5	100% (9/9)	0/10	0/5	0% (0/10)	0/9	0/9	0/5	0% (0/9)
22	HNZC3	Jinghong	Layer	2/5	2/5	2/5	40% (2/5)	0/5	0/5	0% (0/5)	0/5	0/5	0/5	0% (0/5)
23	HNYY2	Jinghong	Layer	0/5	0/5	0/5	40% (2/5)	0/5	0/5	0% (0/5)	0/5	0/5	0/5	0% (0/5)
24	HNPDS	Hyline	Layer	0/6	1/6	1/5	16.7% (1/6)	1/6	1/5	16.7% (1/6)	0/6	0/5	0/5	0% (0/6)
25	HNLY2	Jinghong	Layer	3/6	4/6	3/5	100% (6/6)	0/6	0/5	0% (0/6)	0/6	0/5	0/5	0% (0/6)
26	HNZC4	Hyline	Layer	6/8	8/8	5/5	100% (8/8)	0/8	1/5	12.5% (1/8)	0/8	0/5	0/5	0% (0/8)
27	HNSX	Jinghong	Layer	5/5	5/5	5/5	100% (5/5)	0/5	0/5	0% (0/5)	0/5	0/5	0/5	0% (0/5)
28	HNZC5	Jinghong	Layer	4/7	5/7	3/5	71.4% (5/7)	0/7	0/5	0% (0/7)	0/7	0/7	0/5	0% (0/7)
29	HNFQ	Hyline	Layer	2/11	1/11	1/5	18.2% (2/11)	1/11	2/5	18.2% (2/11)	0/11	0/11	0/5	0% (0/11)
30	HNWS	Muyuan Red	Layer	2/20	3/20	3/5	15% (3/20)	18/20	5/5	90% (18/20)	0/20	0/20	0/5	0% (0/20)
Total							69.5% (203/292)			14.4% (42/292)				4.7% (13/277)
